# HHBSNet: a global channel–spatial attention and multi‐scale dilated convolution network for automatic melasma segmentation

**DOI:** 10.3389/fphys.2025.1665138

**Published:** 2025-11-05

**Authors:** Shange Wang, Lin Xu, Linshuai Zhang, Yujie Zhang, Chen Li, Marcin Grzegorzek, Jing Guo, Tao Jiang

**Affiliations:** ^1^ School of Intelligent Medicine, Chengdu University of Traditional Chinese Medicine, Chengdu, China; ^2^ College of Medicine and Biological Information Engineering, Northeastern University, Shenyang, China; ^3^ Institute of Medical Informatics, University of Lübeck, Lübeck, Germany; ^4^ Chengdu University of Traditional Chinese Medicine, Chengdu, China

**Keywords:** melasma, deep learning, image segmentation, attention mechanism, automatic segmentation

## Abstract

**Objective:**

Melasma is a common acquired facial hyperpigmentation disorder characterized by symmetrical brown patches, often occurring in the zygomatic region, forehead, and upper lip. Its blurred boundaries, color similarity to normal skin, and irregular morphology—combined with lighting variability and skin reflections—pose significant challenges for automated lesion segmentation. This study aims to develop an effective and lightweight deep learning model tailored for accurate melasma segmentation.

**Methods:**

We propose a novel lightweight segmentation network, HHBSNet, specifically designed for melasma lesion analysis. The model incorporates a Global Channel-Spatial Attention (GCSA) module that jointly leverages channel and spatial attention to suppress lighting interference and enhance feature discrimination in low-contrast, irregular boundaries. In addition, a Multiscale Cavity Fusion (MCF) module is introduced to extend the receptive field via multi-dilation rates, enabling effective capture of lesions at various scales without reducing resolution. The network further integrates local-global semantic fusion and adopts a combined loss strategy of cross-entropy and focal loss to address class imbalance.

**Results:**

HHBSNet was evaluated on a self-constructed dataset comprising 501 practical facial melasma images. Quantitative results demonstrate that HHBSNet outperforms existing mainstream segmentation methods, achieving a mean Intersection over Union (Miou) of 79.69%, accuracy (ACC) of 96.68%, F-score of 88.10%, recall of 88.18%, and precision of 87.80%.

**Conclusion:**

The proposed HHBSNet demonstrates superior segmentation performance and robustness in handling melasma’s challenging visual characteristics. Its lightweight structure and strong generalization ability suggest promising potential for application in computer-aided diagnosis and large-scale clinical screening of facial pigmentary disorders.

## 1 Introduction

Melasma is a common skin pigmentation disorder affecting millions of people worldwide (Grimes, 1995; Sheth and Pandya, 2011; Tamega et al., 2013; Handel et al., 2014). It can occur in any gender, but is more common in women (Goh and Dlova, 1999), with men comprising approximately 10% of reported cases (Parish, 2011). Foreign studies have shown that melasma is most commonly seen in patients with Fitzpatrick skin types IV-VI, especially in areas with high UV radiation intensity, such as Asians, Hispanic Latinos, and African-Americans (Grimes, 1995; Sanchez et al., 1981; Taylor, 2003; Pandya and Guevara, 2000). Although there are usually no self-conscious symptoms in clinical practice, melasma has caused great distress to countless patients due to its disfiguring nature, leading to worry, anxiety, and even low self-esteem and depression, which may lead to suicidal tendencies in severe cases. Therefore, melasma has become an important issue of concern to both the medical and cosmetic fields. As a chronic and recurrent disease, melasma has a exerts a profound negative impact on patients’ quality of life, even more than other skin diseases such as acne and rosacea (Balkrishnan et al., 2003). Similar to other skin diseases such as skin cancer (Siegel et al., 2023), the treatment of melasma needs to be predicated on precise lesion segmentation for efficient treatment. In addition, precise lesion segmentation is also a prerequisite for assessing treatment efficacy and disease severity (Liang et al., 2017a).

Skin lesion segmentation has traditionally relied on classical image processing techniques, such as threshold segmentation (Green et al., 1994; Emre Celebi et al., 2013; Celebi et al., 2008; Arsalan et al., 2020) and edge detection, as well as machine learning methods like active contour modeling and support vector machines (Zortea et al., 2011). However, these methods often necessitate complex image pre-processing and post-processing, particularly when the contrast between the lesion and normal skin is low. This can lead to imprecise segmentation boundaries, thereby affecting diagnosis. In response to these challenges, researchers have explored deep Convolutional Neural Network (CNN)-based segmentation algorithms, which have demonstrated significant potential in medical image segmentation due to their ability to enhance segmentation accuracy without the need for complex pre-processing steps. For example, Long et al. (2015) introduced an FCN-based segmentation method that can process input images of arbitrary sizes and has an optimized network structure to reduce redundancy and improve computational efficiency. Despite its strong generalization ability, FCN may sometimes sacrifice image details, indicating room for further optimization of segmentation accuracy. Building on this foundation, Ronneberger et al. (2015) proposed the U-Net model, which comprises an encoder and a decoder. U-Net’s core strength lies in its efficient use of global positional and contextual information, enabling good training results even with limited samples. It has been widely used in precise segmentation tasks.

Further advancements were made by SkinNet (Vesal et al., 2018), which introduced inflated convolution in the encoder to enlarge the convolution kernel’s receptive field, thereby enhancing the network’s ability to capture contextual information and improving segmentation of complex structures. Gu et al. (2019) developed CE-Net, which preserves subtle spatial information features through its feature encoding, context extraction, and feature decoding modules. More recently, Tong et al. (2021) proposed ASCU-Net, which incorporates a triple-attention mechanism to help the network focus on key lesion features, thereby improving segmentation accuracy and recognition performance.

Accurate segmentation of melasma lesions is crucial for clinical diagnosis and treatment; however, current research still faces several significant challenges. First, the confusion between spots and skin texture makes it difficult to accurately distinguish skin lesions. Second, melasma often has irregular shapes and boundaries, which increases the complexity of segmentation algorithms. Third, variations in lighting and reflection phenomena interfere with image processing, affecting segmentation results. Additionally, the labeling process is typically cumbersome and time-consuming, requiring substantial manual intervention. These challenges limit the effectiveness of existing methods in melasma segmentation. Despite significant advancements in the field of medical image segmentation through deep learning methods, handling complex skin lesion images remains a challenge. For instance, studies employing U-Net for melasma segmentation have successfully facilitated the assessment of pigmented skin diseases and supported the development of personalized treatment plans (Liang et al., 2017a; Liang et al., 2017b; Shilaskar et al., 2024; Arsalan et al., 2019; Awais et al., 2021). However, these methods are often limited when dealing with complex lesion images and cannot provide sufficiently accurate segmentation results. While these studies have to some extent propelled the development of melasma segmentation technology, they still fall short in addressing the aforementioned challenges.

1. Therefore, in this study, a more effective image segmentation method, HHBSNet, was developed to improve the accuracy of melasma lesion segmentation and thus provide more reliable support for clinical diagnosis and treatment. Our model includes the following contributions:
2. Global Channel-Spatial Attention (GCSA) module: Existing methods often struggle to effectively differentiate between lesion areas and normal skin when dealing with complex backgrounds and noise. To enhance the model’s ability to focus on lesion regions, we have designed a Global Channel-Spatial Attention module. This module integrates channel attention, channel shuffling, and spatial attention mechanisms to capture global dependencies within the feature map. In this way, the model can better focus on important features while suppressing irrelevant noise information. This not only improves segmentation accuracy but also enhances the model’s robustness against complex backgrounds.
3. Multi-scale Cavity Fusion (MCF) module: Traditional methods often fail to accurately capture contextual information when dealing with lesion areas that have complex shapes and boundaries due to limited receptive fields. Our MCF module significantly enlarges the receptive field by setting different dilation rates while maintaining image resolution. This allows the model to capture a broader range of contextual information, which is particularly effective in processing melasma images with irregular shapes and boundaries, thereby improving segmentation accuracy.
4. Global feature integration: Existing methods often fall short in handling global and local information, leading to less accurate segmentation results. Our network incorporates global average pooling to extract global features of the image and combines this global information with local features. This design helps the network better understand the relationship between different parts of the image and the whole, further enhancing the model’s segmentation performance for skin lesions.

To verify the effectiveness of the method in this paper, we conducted experiments on a privately collected melasma image dataset. The experimental results show that on this melasma segmentation dataset, our method is competitive in performance and outperforms existing commonly used and State-Of-The-Art (SOTA) methods. This suggests that HHBSNet can effectively address the challenges in melasma segmentation and provide a more accurate and efficient tool for clinical diagnosis and treatment.

The remaining sections of this work are organized according to the following structure: Section 2 summarizes the current state of the art in skin lesion segmentation research, sorting out the trends and challenges of current research. Section 3 details the network design proposed in this study, focusing on its core technologies and innovations. Section 4 verifies the performance and effectiveness of the proposed method through experiments and analyzes it in comparison with existing techniques. In Section 5, based on the experimental findings in Section 4, the proposed method is more comprehensively analyzed and summarized, and the existing problems are pointed out and potential improvement directions are proposed. In addition, the abbreviations used in the paper are shown in Table 1.

**TABLE 1:** Symbols and abbreviations used in the article.

No.	Full name	Abbreviation
1	Global Channel-Spatial Attention	GCSA
2	Multi-scale Cavity Fusion	MCF
3	Convolutional Neural Network	CNN
4	Fully Convolutional Network	FCN
5	Context Encoder Network	CENet
6	State-Of-The-Art	SOTA
7	Squeeze-and-Excitation Networks	SENet
8	Vision Transformer	ViT
9	Coordinate Attention	CA
10	Efficient Channel Attention Module	ECA-Net
11	Multilayer perceptron	MLP
12	Batch Normalization	BN
13	Stochastic Gradient Descent	SGD
14	Mean Intersection over Union	MIoU
15	Accuracy	ACC
16	Mean Recall	MRecall
17	Receiver Operating Characteristic	ROC
18	False Positive Rate	FPR
19	Ground Truth	GT
20	HHB	Melasma lesions
21	LF	The left facial area
22	RF	The right facial area
23	XB	The chin area
24	ET	The forehead area

## 2 Relate work

### 2.1 Skin disease segmentation

Skin lesion segmentation is a crucial step in the diagnosis and treatment of skin diseases. Accurate segmentation of the lesion area aids physicians in quantitative analysis of the lesion, monitoring its progression, and evaluating treatment efficacy. Traditional methods primarily rely on handcrafted features, while the advent of deep learning has brought significant changes to this field in recent years.

In the early research of skin lesion segmentation, traditional methods mainly relied on handcrafted features. Although these methods can achieve segmentation goals to some extent, they have obvious limitations, especially when facing diverse lesion types, in terms of scalability and adaptability. Celebi et al. (2009) proposed a histogram thresholding method based on the intensity distribution. This method determines the segmentation threshold by analyzing the grayscale histogram of the image to distinguish between the lesion area and normal skin. However, it is sensitive to lighting conditions and noise and struggles with complex grayscale distributions in lesion areas. Otsu (1979) introduced a variance-based thresholding method, which determines the optimal threshold by maximizing the between-class variance of the foreground and background. Otsu’s method, known for its simplicity and efficiency, has been widely used in automated medical image segmentation. However, when the grayscale difference between the lesion area and the background is not significant, the segmentation effect is compromised. Peruch et al. (2013) enhanced segmentation robustness through noise suppression and post-processing techniques. They employed filtering algorithms to remove noise and optimized segmentation results through morphological operations and other post-processing steps. Although these methods improved segmentation accuracy to some degree, they rely on prior knowledge of noise characteristics, and the post-processing steps increased computational complexity. Patiño et al. (2018) introduced superpixel merging with color invariance to improve robustness against illumination changes. This method divides the image into superpixels and then merges them based on color and texture features to achieve segmentation. While it addresses the issue of uneven lighting to some extent, the superpixel merging process is complex and less adaptable to variations in the shape and size of lesion areas. These traditional methods, though effective in certain specific scenarios, gradually reveal their limitations when dealing with complex skin lesion images, especially in the segmentation of melasma. Melasma lesion areas often have complex textures and irregular boundaries, which are difficult for traditional methods to segment accurately.

With the rise of deep learning technology, the field of skin lesion segmentation has witnessed a significant transformation. Deep learning methods, which automatically learn image features, have overcome the limitations of traditional methods and significantly enhanced segmentation accuracy and robustness. Long et al. (2015) introduced the Fully Convolutional Networks (FCNs), a pioneering work of deep learning in image segmentation. FCNs replace the fully connected layers of Convolutional Neural Networks (CNNs) with convolutional layers to achieve pixel-level prediction, providing an end-to-end solution for image segmentation. Ronneberger et al. (2015) proposed the U-Net, a classic medical image segmentation network that employs an encoder-decoder architecture with skip connections to preserve spatial resolution. U-Net has shown excellent performance in processing medical images, especially in extracting lesion areas from small targets and complex backgrounds. DeepLab (Chen et al., 2017) utilizes atrous convolutions to expand the receptive field, thereby better capturing contextual information in the image. This method excels in handling targets with complex shapes and boundaries, effectively reducing boundary blurring issues. UNet 3+ (Huang et al., 2020) further improves the U-Net architecture by integrating full-scale skip connections to enhance feature fusion, thereby increasing segmentation accuracy and robustness. This method performs particularly well in processing lesion areas with multi-scale features. Yuan et al. (2017) enhanced segmentation performance by introducing batch normalization and customized loss functions. These techniques help accelerate network convergence and improve model adaptability to different lesion types. Dai et al. (2022) developed multi-scale residual modules to address the structural variability in lesions. These modules effectively capture features at different scales, thereby improving segmentation accuracy. These deep learning methods have shown excellent performance in processing complex skin lesion images, especially in melasma segmentation, where they can better handle the complex textures and irregular boundaries of lesion areas.

To tackle the unique challenges in skin lesion segmentation, researchers have developed a series of specialized models and methods. BAT (Festa et al., 2023) applies deformable convolutions to reduce boundary ambiguity, thereby improving segmentation accuracy. Experiments have shown that this method can reduce boundary ambiguity by 34%, significantly enhancing the quality of segmentation results. MSCA-Net (Sun et al., 2023) enables real-time inference through multi-scale coordinate attention, making it suitable for deployment on resource-constrained platforms. This method maintains high segmentation accuracy while significantly reducing computational resource requirements. DC-Net (Wang et al., 2022) leverages contrastive learning to differentiate between malignant melanoma and benign lesions. This method improves the recognition ability of different lesion types by learning the feature differences between them. These specialized models and methods have shown excellent performance in processing complex skin lesion images, especially in melasma segmentation, where they can better handle the complex textures and irregular boundaries of lesion areas.

The advent of deep learning has brought significant changes to the field of skin lesion segmentation. From traditional methods that rely on handcrafted features to current deep learning methods that automatically learn features, segmentation technology has seen a significant increase in accuracy and robustness. These methods not only better handle complex backgrounds and multi-scale features in lesion areas but also adapt to the diverse needs of different lesion types. Moreover, the development of specialized models and methods for skin lesion segmentation has further improved segmentation precision and efficiency. With the continuous development of technology, more efficient, accurate, and robust skin lesion segmentation methods are expected to be developed in the future, providing strong support for the diagnosis and treatment of skin diseases. In the field of melasma segmentation, further research and application of these methods will help improve the diagnostic accuracy and treatment efficacy of melasma.

### 2.2 Attention mechanisms in segmentation

Attention mechanism has become a key technique to enhance the performance of medical image segmentation, especially in tasks such as dealing with complex textures, lesions with different sizes or fuzzy boundaries, etc. It shows significant advantages. Its applications range from spatial enhancement to global semantic modeling, effectively improving the accuracy of segmentation.

Squeeze-and-Excitation Networks (SENet) (Hu et al., 2018) introduces a feature recalibration mechanism in the channel dimension, which improves the ability of the model to select effective features, while CBAM (Woo et al., 2018) introduces a spatial attention module, which can help to localize the lesion area more accurately, and Attention U-Net (Oktay et al., 2018) introduces a spatial attention gating mechanism, which can be used for the segmentation of complex textures with different sizes or ambiguous boundaries. Attention U-Net improves the Dice coefficient by 8.7% in the pancreas segmentation task by using the spatial attention gating mechanism, which proves its effectiveness in removing irrelevant regions. MA-Net (Dharejo et al., 2022) and MA-UNet (Cai and Wang, 2022) merge multi-scale and multi-dimensional attention mechanisms to enable the model to extract both global context and local detail features at the same time. These hybrid strategies significantly enhance the recognition of lesion boundaries in complex scenarios. The SCSE module (Roy et al., 2018) combines both spatial and channel attention to effectively suppress background noise and enhance the robustness of feature representation. PraNet (Fan et al., 2020) introduces the inverse attention mechanism to gradually optimize the prediction of fuzzy regions, and reduces the false-positive rate by 15% in polyp segmentation, and demonstrates better accuracy in the difficult-to-segment regions. Regions that are difficult to segment.

Vision Transformer (ViT) (Dosovitskiy et al., 2020) introduced the self-attention mechanism in computer vision for the first time, and realized the modeling of remote dependencies. A series of derived structures based on this (e.g., TransUNet (Chen et al., 2021), MedT (Valanarasu et al., 2021), Swin-UNet (Cao et al., 2022)) introduced the global context modeling capability into medical image segmentation tasks. TransAttUNet (Chen et al., 2023) combines multilevel attention with Vision Transformer and achieves on the ISIC 2018 data set a 92.1% Coordinate Attention (CA) (Hou et al., 2021) with Efficient Channel Attention Module (ECA-Net) (Wang et al., 2020) introduces an efficient attention mechanism that maintains a strong representation capability while keeping a low computational effort, which is suitable for real-time or resource-constrained scenarios.

In conclusion, the development of attention mechanisms, from lightweight design to Transformer-based global modeling, continues to push the performance ceiling of medical image segmentation tasks.

## 3 Methods

We employ MobileNetV2 (pre-trained on ImageNet) as the backbone feature extractor due to its lightweight depthwise separable convolutions and strong representation capability. The backbone extracts multi-scale feature maps at four resolution levels.

**FIGURE 1:**
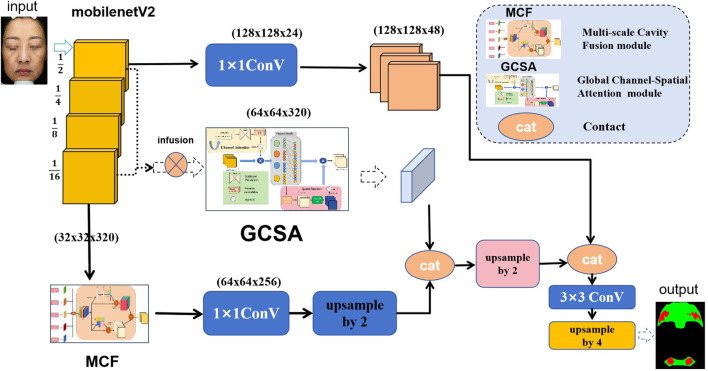
Diagram of the overall framework of the HHBSNet model.

Figure 1 demonstrates the three core modules of the HHBSNet framework: the GCSA module, the MCF module and the Global feature fusion function; the GCSA module: through channel attention, channel shuffling, and spatial attention working in concert, the global dependencies in the feature map are captured to effectively highlight the speckle region and suppress background noise. And the Infusion block extracts first and final features from the MobileNetV2 backbone and fuses them via concatenation followed by a (1 × 1) convolution. This allows the network to combine shallow texture details with high-level semantic information before further processing in the GCSA module. MCF module: Separable convolution with different cavity rates is used to significantly expand the receptive field without loss of resolution in order to simultaneously capture lesion edge details and wide-area contextual information. Global feature fusion: global average pooling is used to extract the overall semantics of the image, and global information is fused with local features to enhance the network’s overall perception of melasma morphology and distribution.

### 3.1 Global channel-spatial attention module

**FIGURE 2:**
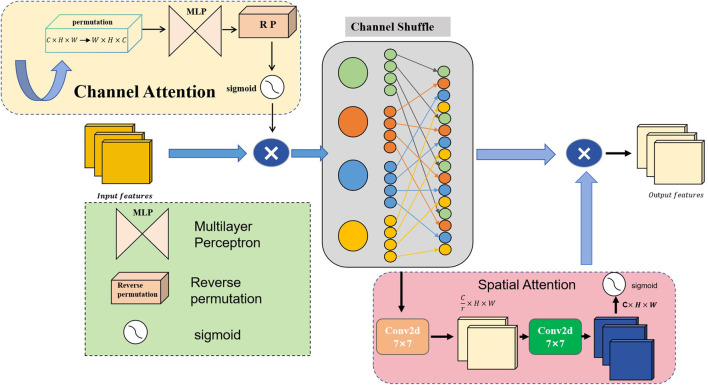
Schematic of the global channel-spatial attention (GCSA) structure.

In this study, the GCSA is designed to enhance the representation of input feature maps. The module combines channel attention, channel shuffling, and spatial attention mechanisms designed to capture global dependencies in feature maps. The specific flowchart is shown in Figure 2 below, and the detailed step-by-step explanations are given below.

The input feature map $F_{\text{input}}\in R^{C\times H\times W}$ , consisting of C channels of spatial size H×W , is first fed into the channel-attention submodule and then into the spatial-attention submodule. Within the channel-attention submodule, $F_{\text{input}}$ is first permuted from shape C×H×W to W× H×C . lt then passes through two successive Multilayer perceptron (MLP) layers to capture inter-channel dependencies: the first MLP reduces the channel dimension to $\frac{C}{4}$ , followed by a ReLU activation, and the second MLP restores the channel dimension to C . After a reverse permutation back to C×H×W and a Sigmoid activation, the channel-attention map is generated. Elementwise multiplication with the original input yields the enhanced feature map. As shown in Equation 1:

$$F_{\text{channel}}=\sigma \left(\text{MLP}\left(\text{Permute}\left(F_{\text{input}}\right)\right)\right)⊙F_{\text{input}},$$ (1)

where σ denotes the Sigmoid function, ⊙ denotes element-wise multiplication, and $F_{\text{channel}}$ is the resulting channel-refined feature map.

To further mix and share information across channels, we apply the Channel Shuffle operation. The enhanced feature map $F_{\text{channel}}$ is divided into 4 groups of $\frac{C}{4}$ channels each; within each group, channels are transposed to shuffle their order, and then all groups are concatenated back to the original shape C×H×W. As shown in Equation 2:

$$F_{\text{shuffle}}=\text{ChannelShuffle}\left(F_{\text{channel}}\right),$$ (2)

where $F_{\text{shuffle}}$ is the shuffled feature map.

In the spatial-attention submodule, $F_{\text{shuffle}}$ first passes through a 7×7 convolution that reduces its channel dimension to $\frac{C}{4}$ , followed by Batch Normalization (BN) and ReLU activation. A second 7×7 convolution then restores the channel dimension to C , followed by another BN. A Sigmoid activation generates the spatial-attention map, which is multiplied element-wise with $F_{\text{shuffle}}$ to produce the final output. As shown in Equation 3:

$$F_{\text{spatial}}=\sigma \left(\text{Conv}\left(\text{BN}\left(\text{ReLU}\left(\text{Conv}\left(F_{\text{shuffle}}\right)\right)\right)\right)\right)⊙F_{\text{shuffle}},$$ (3)

where $F_{\text{spatial}}$ is the spatially refined feature map.

The module’s output $F_{\text{out}}=F_{\text{spatial}}$ thus contains features enhanced by channel attention, channel shuffling, and spatial attention, ready to be passed to the downstream segmentation head.

### 3.2 Multi-scale cavity fusion module

**FIGURE 3:**
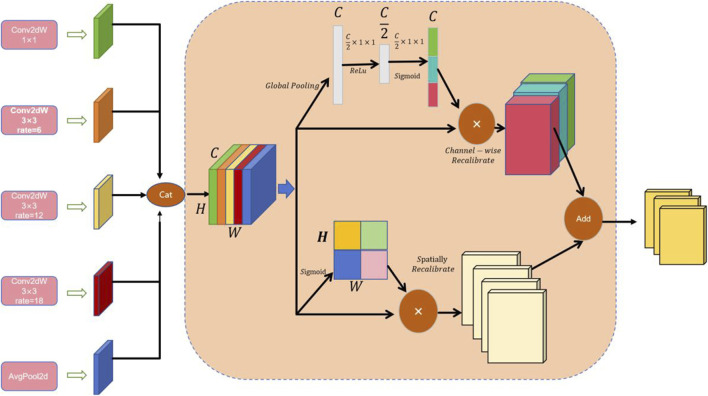
Schematic of the structure of the multiscale cavity fusion module (MCF).

By integrating channel and spatial attention mechanisms and using depthwise separable convolutions with different dilation rates, the MCF module seeks to improve feature representation. For complicated visual tasks like semantic segmentation and object detection, this architecture is critical for gathering both extensive and detailed contextual information in images. Figure 3 provides an illustration of the architecture. Firstly, our MCF architecture employs five parallel convolutional branches for multi-scale feature extraction; each branch is configured with a different dilation rate to expand the receptive field and capture varying spatial information. The first branch uses a 1×1 convolution kernel to maintain the spatial scale and directly extract features. The second branch utilizes a 3 × 3 convolution kernel with a dilation rate of 6 to moderately expand the receptive field. The third branch employs a 3 × 3 convolution kernel with a dilation rate of 12 to further expand the receptive field and capture broader contextual information. The fourth branch uses a 3 × 3 convolution kernel with a dilation rate of 18 to provide the widest receptive field. The fifth branch applies global average pooling to extract global contextual features, enhancing the model’s understanding of the overall layout. After these processes, the feature maps have the shape H×W×C. The Concat function is used to concatenate the different outputs along the channel dimension, resulting in a comprehensive feature map. Let the input feature map be $X\in R^{H\times W\times C}$ , where H,W and C are the height, width, and number of channels, respectively. The outputs of the five branches can be mathematically described as follows. As shown in Equations 4–8:

$$OP_{1}=f_{1\times 1Conv}\left(X\right)$$ (4)

$$OP_{2,3,4}=f_{3\times 3ConvDW}\left(X,r=\left[6,12,18\right]\right)$$ (5)

$$OP_{5}=f_{upsample}\left(f_{1\times 1conv}\left(f_{avg-pool}\left(X\right)\right)\right)$$ (6)

$$F_{feature}=\text{Concat}\left(OP_{1},OP_{2},OP_{3},OP_{4},OP_{5}\right)$$ (7)

$$Output=f_{1\times 1Conv}\left(F_{feature}\right)$$ (8)

where X denotes the input feature map with dimensions H×W×C , and $OP_{i}$ signifies the outcome of each distinct operation, r is the dilation ratio, and $f_{avg-pool}$ is the average pooling operation. The concatenation of various outputs in the channel dimension is known as the Concat function. Lastly, the final output feature map’s shape remains H×W×C. Two different kinds of attention mechanisms are then used to calibrate the combined feature map. To get the global features for each channel, the channel attention mechanism first applies global average pooling to the combined feature map. It then uses two fully linked layers with Sigmoid and ReLU activation functions, respectively, to learn the importance weights for each channel. Channel weighting is achieved by multiplying these weights by the original feature map on a per-channel basis. As shown in Equations 9–11.

$$F_{a\nu g}=\frac{1}{H\times W}\sum_{i=1}^{H}\sum_{j=1}^{W}F\left(i,j\right)$$ (9)

$$w_{c}=\sigma \left({\text{FC}}_{2}\left(\text{ReLU}\left({\text{FC}}_{1}\left(F_{a\nu g}\right)\right)\right)\right)$$ (10)

$$F_{c}^{\prime }=w_{c}⊙F$$ (11)

where $F\left(i,j\right)$ represents the value of the feature map at position $i,j,{\text{FC}}_{1}$ and FC_2_ are the two fully connected layers, σ denotes the Sigmoid activation function, and $w_{c}$ is the importance weight for each channel. ⊙ represents element-wise multiplication, and $F_{c}^{\prime }$ is the feature map after channel weighting. To create the spatial feature map, the spatial attention mechanism applies global pooling to the combined feature map along the channel dimension. It then uses a Sigmoid activation function and a 1 ×1 convolution to learn the importance weights for every spatial position. Spatial weighting is achieved by multiplying these weights element-wise by the original feature map. As shown in Equations 12–14.

$$F_{spatial}=\left[AvgPool\left(F\right),MaxPool\left(F\right)\right]$$ (12)

$$w_{s}=\sigma \left(Conv_{1\times 1}\left(F_{spatial}\right)\right)$$ (13)

$${F_{s}}^{\prime }=w_{s}⊙F$$ (14)

where $\left[\cdot ,\cdot \right]$ denotes the concatenation operation, resulting in the spatial feature map $F_{spatial}$ , and ${F_{s}}^{\prime }$ is the feature map after spatial weighting. Finally, the output feature maps from the channel attention and spatial attention mechanisms are fused through element-wise addition. The fused feature map is then combined with the original merged feature map through element-wise summation to integrate and enhance the relevant features. The enhanced feature map is subsequently processed through a 1×1 convolution layer for dimensionality reduction and integration, resulting in the final output feature map $F_{output}$ . As shown in Equations 15–16.

$$F_{fused}={F_{c}}^{\prime }+{F_{s}}^{\prime }$$ (15)

$$F_{output}=Conv_{1\times 1}\left(F_{fused}\right)$$ (16)

where $F_{fused}$ is the feature map obtained by fusing the channel and spatial features after weighting.

## 4 Experience

### 4.1 Dataset

The melasma image dataset used in this study was collected in 2023 at the outpatient dermatology clinic of the Affiliated Hospital of Chengdu University of Traditional Chinese Medicine. It includes 501 patients (aged 18–65 years) who were clinically diagnosed with melasma. At the early stage of data collection, we intentionally aimed to include a roughly balanced male-to-female ratio in order to mitigate potential gender bias and allow the model to generalize to male patients as well. However, as the dataset expanded, the majority of cases were contributed by female patients, which is consistent with the known epidemiology of melasma (around 90% female). Thus, while our dataset contains a higher proportion of male patients than the general clinical prevalence, female patients still dominate the final dataset.

**FIGURE 4:**
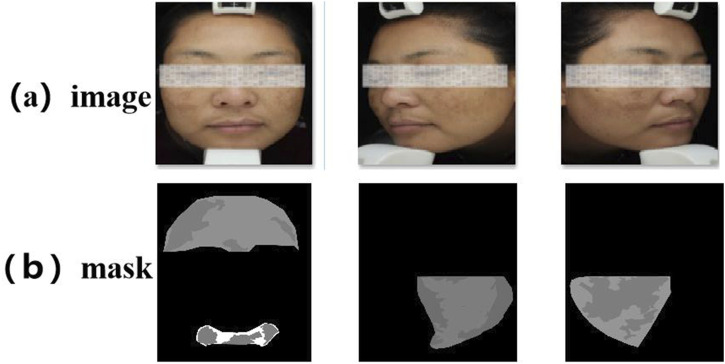
Example diagram of the melasma dataset. **(a)** Raw facial image captured by the VISIA multispectral system. **(b)** Expert-labeled melasma lesion masks.

All participants provided written informed consent. To ensure consistent lighting and color temperature, all images were captured under standardized conditions using a VISIA multispectral skin imaging system (Canfield Scientific, United States) in a cold-light environment. During image acquisition, the camera was maintained at a fixed distance of 30 cm from the participant’s face. After the raw data were initially cleaned and blurred, overexposed or heavily reflected images were excluded, the melasma lesion areas were independently labeled by a dermatologist with extensive clinical experience using the LabelMe tool, and were uniformly cropped to a size of 512 × 512, as shown in Figure 4 below. In order to ensure fairness of the experiment, this dataset was divided into training, validation, and test sets according to an 8:1:1 ratio (corresponding to 401, 50, and 50 images, respectively). The dataset will be made available upon reasonable request for academic and non-commercial research purposes, subject to obtaining appropriate ethical approval.

### 4.2 Experience details

All experiments were implemented using the PyTorch framework and conducted on a workstation equipped with an NVIDIA GeForce RTX 3080 GPU. Input images were uniformly resized to 512 × 512 pixels. Each model was trained for 180 epochs with a batch size of 8. We used Stochastic Gradient Descent (SGD) as the optimizer, with a momentum of 0.9 and an initial learning rate of 7 × 10^−3^, which was decayed according to a step-based schedule to facilitate convergence. In order to improve generalization and robustness, we applied on-the-fly data augmentation, including random horizontal flipping, random vertical flipping, and random cropping during training.

### 4.3 Evaluation metrics

To comprehensively assess segmentation performance on the melasma dataset, we employed seven metrics: Mean Intersection over Union (MIoU), pixel-level Accuracy (ACC), F1 Score, Mean Recall (MRecall), Precision, Dice coefficient, and Specificity. Specifically: MIoU measures the spatial overlap between predictions and ground truth; ACC is the ratio of correctly classified pixels to total pixels; the F1 Score is the harmonic mean of Precision and Recall, reflecting balanced performance; MRecall represents the average recall across all positive (lesion) pixels; Precision is the proportion of predicted lesion pixels that are truly lesions; the Dice coefficient quantifies the similarity between predicted and true lesion regions and is particularly sensitive to small lesions; Specificity measures the proportion of correctly classified background pixels, reflecting the model’s ability to avoid false positives.

### 4.4 Loss function

Given the class imbalance inherent in skin lesion segmentation, we combined the Cross-Entropy Loss (Creswell et al., 2017) with Focal Loss (Lin et al., 2017) to form a composite objective. The relationship is described by Equations 17, 18:

$$L=\lambda L_{\text{CE}}+\left(1-\lambda \right)L_{\text{FL}},$$ (17)

where

$$L_{\text{CE}}=-\sum_{i}y_{i}\log\left(p_{i}\right),\;L_{\text{FL}}=-\alpha {\left(1‐p_{i}\right)}^{\gamma }y_{i}\log\left(p_{i}\right)$$ (18)

where $y_{i}$ is the ground-truth label and $p_{i}$ is the predicted probability for pixel i ; α balances positive and negative examples; γ focuses training on hard-to-classify samples; and $\lambda \in \left[0,1\right]$ controls the weighting between the two losses. This design leverages the stable convergence of cross-entropy and the hard-example emphasis of Focal Loss, thereby enhancing overall segmentation accuracy and robustness.

### 4.5 Analysis of experimental results

With the aim of validating the proposed HHBSNet in facial melasma segmentation tasks, we conducted comparative experiments on several mainstream segmentation models. Table 2 summarizes the segmentation performance of HHBSNet and baseline models on facial melasma lesions. In addition to standard metrics (mIoU, ACC, Precision, Recall, and F1-score), we report Dice coefficient and specificity to provide a more comprehensive evaluation. Statistical error margins (mean ± standard deviation over 5 runs) are included to demonstrate performance stability. HHBSNet achieves the highest mIoU (0.7889 ± 0.0016), ACC (0.9659 ± 0.0003), F1-score (0.8778 ± 0.0009), and Precision (0.8777 ± 0.0013), indicating robust and accurate lesion segmentation. It is noted that the Dice coefficient for HHBSNet is lower compared to some baseline models. This is primarily due to the small relative size of facial melasma lesions compared to the overall facial area, which amplifies the impact of even minor segmentation errors on the Dice score. Meanwhile, HHBSNet maintains high specificity (0.9467 ± 0.0001), confirming that the model effectively avoids false positives in the large background area. Therefore, despite the relatively low Dice value, HHBSNet demonstrates superior overall segmentation performance on facial melasma lesions.

**TABLE 2:** Segmentation performance of HHBSNet and baseline models on facial melasma lesions.

Model	MIoU	ACC	Precision	Recall	F1	Dice	Specificity
U-Net	0.6656 ± 0.0009	0.9373 ± 0.0002	0.7285 ± 0.0006	0.8742 ± 0.0008	0.7947 ± 0.0006	0.4236 ± 0.0033	0.9644 ± 0.0003
U-Net++	0.6660 ± 0.0021	0.9372 ± 0.0004	0.7284 ± 0.0018	0.8738 ± 0.0017	0.7947 ± 0.0017	**0.4294** ± **0.0044**	0.9644 ± 0.0003
FPN	0.6684 ± 0.0063	0.9367 ± 0.0003	0.7278 ± 0.0019	0.8736 ± 0.0021	0.7955 ± 0.0021	0.4365 ± 0.0072	0.9639 ± 0.0008
MALUNet	0.6681 ± 0.0084	0.9443 ± 0.0031	0.8064 ± 0.0306	0.7930 ± 0.0202	0.7962 ± 0.0083	0.2553 ± 0.0281	0.9341 ± 0.0071
DeepLabV3	0.6679 ± 0.0041	0.9415 ± 0.0021	0.7744 ± 0.0151	0.8246 ± 0.0126	0.7942 ± 0.0040	0.2867 ± 0.0296	0.9425 ± 0.0046
EGEUNet	0.7071 ± 0.0064	0.9490 ± 0.0020	0.8069 ± 0.0193	0.8486 ± 0.0054	0.8255 ± 0.0064	0.2686 ± 0.0046	0.9450 ± 0.0013
DCSAU-Net	0.7132 ± 0.0026	0.9494 ± 0.0004	0.8159 ± 0.0037	0.8480 ± 0.0035	0.8298 ± 0.0019	0.2783 ± 0.0045	**0.9473** ± **0.0010**
HHBSNet (Ours)	0.7889 ± 0.0016	0.9659 ± 0.0003	0.8777 ± 0.0013	0.8781 ± 0.0008	0.8778 ± 0.0009	**0.2316** ± **0.0015**	**0.9467** ± **0.0001**

Metrics include mean Intersection over Union (mIoU), accuracy (ACC), precision, recall, F1-score, Dice coefficient, and specificity. Values are reported as mean ± standard deviation over 5 runs. Due to the small size of lesions relative to the facial area, Dice values may appear lower but do not reflect the overall segmentation quality. Bold numbers indicate the best performance in each column.

**FIGURE 5:**
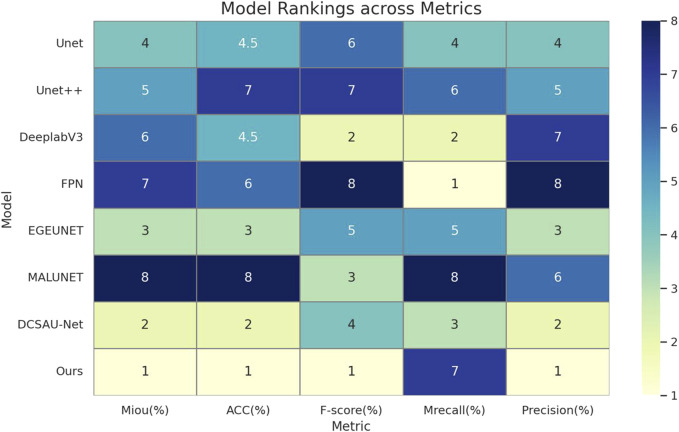
This heatmap of model performance rankings shows the rank of each model on the different evaluation metrics (1 indicates the best): the Ours model ranks first (lightest color) in essentially all metrics, and performs the best.

As can be seen from Table 2 and Figure 5, the median-enhanced spatial and channel attention module introduced by HHBSNet effectively improves the model’s focus on key regions in the feature extraction stage. Channel attention focuses on semantically significant channel features through global pooling operations, while spatial attention strengthens the model’s ability to respond to edge blurring and irregular regions with the help of multi-scale deep convolution. In addition, the HHBSNet enables the model to capture both the local texture details of the lesion boundaries and the integrity of the overall lesion morphology by integrating the low-level, mid-level and high-level semantic features. The experimental results show that although some traditional models (e.g., DeepLabV3 and MALUNET) achieve high values in Recall (90.15% and 88.12%, respectively), their Precision is obviously insufficient (72.03% and 72.51%, respectively), and there are more false detections. On the other hand, HHBSNet maintains a high Recall (88.18%) while significantly improving the Precision, indicating that the model significantly enhances the specificity while ensuring the sensitivity, and effectively suppresses the misidentification of non-lesion regions. Moreover, to assess the robustness of performance improvements, we performed paired t-tests on the MIoU across five repeated runs (see Table 3). HHBSNet showed statistically significant improvements over all baselines (all p < 0.001).

**TABLE 3:** Statistical significance analysis of HHBSNet compared with baseline models on facial melasma lesion segmentation.

Baseline	t-value	p-value	Significance
U-Net	133.42	1.89e-08	p < 0.001
U-Net++	75.33	1.86e-07	p < 0.001
FPN	40.90	2.13e-06	p < 0.001
MALUNET	26.08	1.28e-05	p < 0.001
DeepLabV3	52.78	7.72e-07	p < 0.001
EGEUNet	25.71	1.36e-05	p < 0.001
DCSAU-Net	81.02	1.39e-07	p < 0.001

Paired t-tests were performed on the mIoU scores over 5 independent runs. t-values, p-values, and significance levels are reported. All comparisons show p < 0.001, indicating that HHBSNet’s improvements are statistically significant.

**FIGURE 6:**
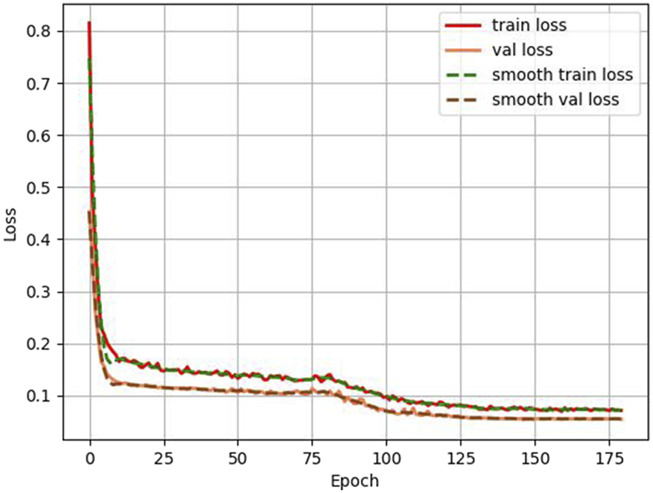
Loss curves (solid lines) and corresponding smoothing curves (dashed lines) on the training and validation sets.

**FIGURE 7:**
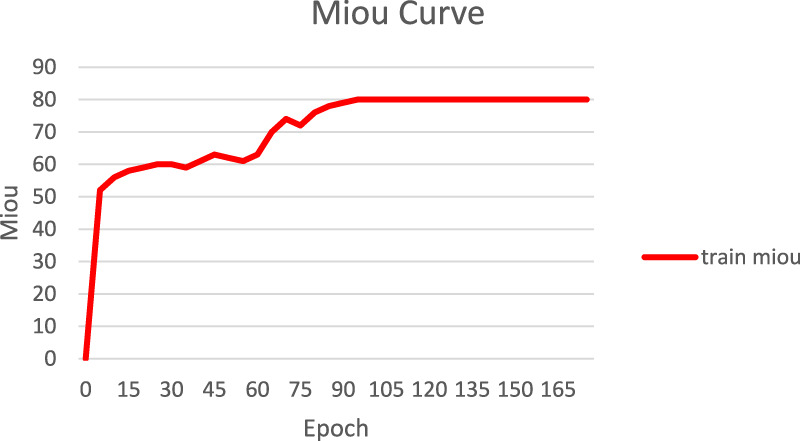
Variation of model MIoU with epoch during training.

Also, in this study, we tracked and recorded the loss variation and segmentation accuracy improvement during model training, as shown in Figures 6, 7. Figure 6 presents the loss curves (red and orange solid lines) and their smoothing curves (green and brown dashed lines) on the training and validation sets. It can be seen that the loss drops rapidly from about 0.8 to within 0.2 at the beginning of training, then enters a slow decline phase between the 20th and 80th epochs and stabilizes after about the 100th epoch, eventually converging to about 0.07–0.06; the validation set loss closely follows the training set loss curve and always remains at a similar level, indicating that the model does not show obvious overfitting during the whole training process. Figure 6 shows the curve of MIoU with epoch during the training process. The model achieves more than 50% MIoU in the first 5 epochs, and thereafter, with the continuous optimization of the network, the MIoU rises smoothly to reach about 70% in the 80th epoch, and further increases to about 78%–80% in the 120th-150th epochs, and finally converges. The smooth rise of this curve is corroborated by the continuous decrease of loss in Figure 7, which fully demonstrates that the designed HHBSNet architecture and loss function combination can improve the melasma segmentation accuracy stably and efficiently.

### 4.6 Visualization and analysis of experimental results

**FIGURE 8:**
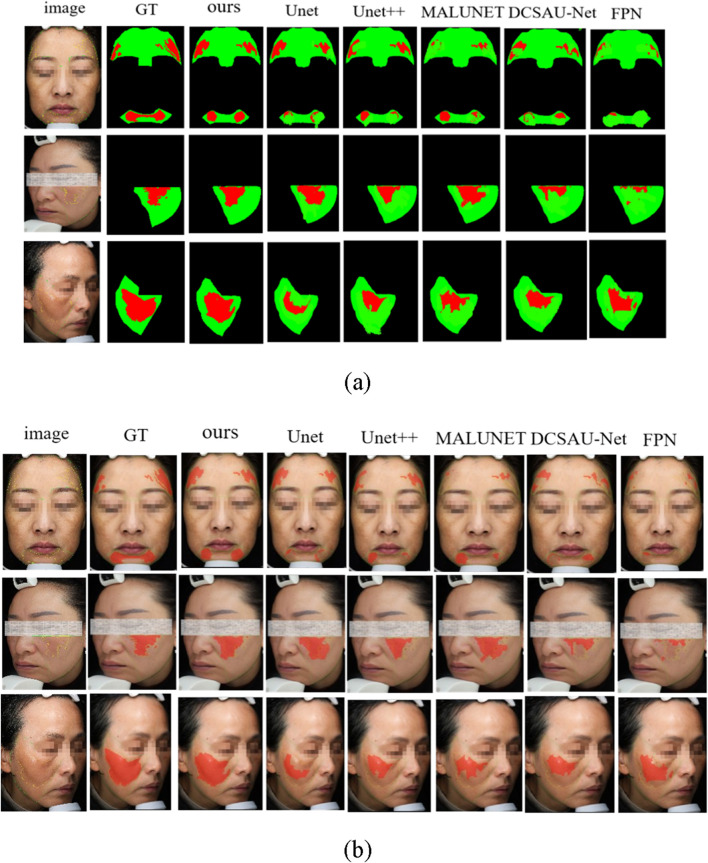
Comparison of melasma segmentation visualization. **(a)** Binary mask results showing lesion areas. **(b)** Lesion boundaries overlaid on the original facial images for improved interpretability. Red indicates lesion boundaries, and green denotes facial contours.

In order to further verify the specific performance of each model in the task of facial melasma segmentation, we selected three groups of representative case images to demonstrate the segmentation results of different methods. Figure 8a shows the visualized comparison diagram, where each row corresponds to one patient respectively, the first column is the original facial image, followed by the segmentation outputs of Unet, Unet++, MALUNET, DCSAU-Net, FPN, and HHBSNet proposed in this paper in that order. In the segmentation diagram, the red region represents the melasma region recognized by the model, green is the normal skin region, and black is the background or unlabeled region. In addition, we superimposed the lesion area onto the original facial image to better display the image content. As shown in Figure 8b.

From the figures, the following points can be observed: the Unet and FPN models have obvious lesion area leakage, especially in the areas with blurred boundaries and uneven illumination, some melasma areas are not recognized, and the overall segmentation results are rough; Unet++ improves in capturing the lesion edges, and is able to recognize some of the lesions with a clearer contour, but there are still artifacts in the areas with a similar color to skin color and a lower contrast ratio. The segmentation accuracy of MALUNET is significantly higher than the previous models, and the model is able to outline the lesions more stably, but there is still the problem of blurring or over-expansion of the boundary of some lesion areas; The DCSAU-Net enhances the coherence and stability of lesion segmentation. However, in some instances, it may exhibit over-segmentation of normal regions, potentially leading to inaccurate segmentation outcomes. The model presented in this study demonstrates superior performance across various comparisons, characterized by smooth edges and distinct structural details within the identified melanoma areas. It also maintains high consistency and accuracy under varying angles, diverse skin tones, and different lighting conditions. Especially in areas with blurred boundaries and dense or sparse spots, its prediction results fit the real lesions more closely, with almost no obvious omissions or misjudgments.

In conclusion, the advantages of the proposed model in maintaining the structural integrity and accuracy of the lesions are further verified from the visual results, which fully demonstrate that the model has stronger clinical adaptability in practical application scenarios.

**FIGURE 9:**
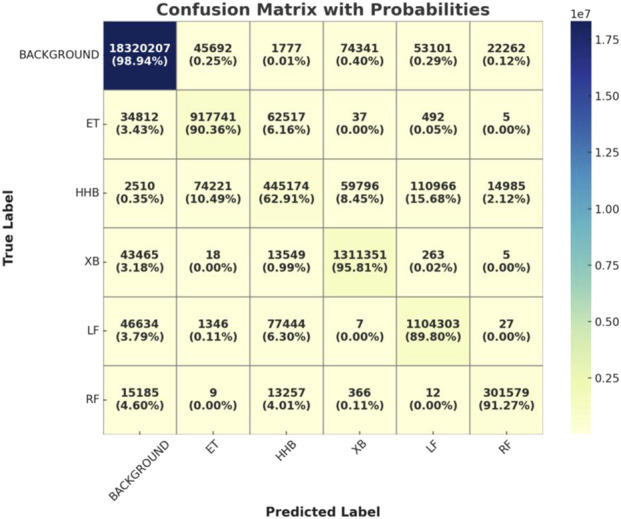
Visualization of the confusion matrix of the HHBSNet model on the test set.

And, to further evaluate the fine-grained performance of the HHBSNet model in the multi-category skin lesion segmentation task, we plotted and analyzed the confusion matrix of the model on the test set. Figure 9 presents the confusion matrix of HHBSNet on the test set, providing insight into its discriminative ability across different categories. The diagonal dominance indicates that the model achieves consistently high classification accuracy, especially for the “BACKGROUND” class, where the correct predictions far exceed other categories. This confirms HHBSNet’s ability to reliably exclude non-lesion regions, which is essential in avoiding false positives in clinical practice. For melasma-related subclasses (“ET,” “HHB,” “XB,” “LF,” “RF”), the model also demonstrates robust performance, with high counts of correct predictions across all categories. While some misclassifications are observed—for instance, “HHB” partially confused with “LF” or “ET”—these errors are attributable to the inherent similarity and boundary ambiguity of these lesion patterns. Importantly, the confusion matrix reveals that HHBSNet achieves balanced recognition across major and minor subclasses, even under challenges such as category overlap and data imbalance, underscoring its strong generalization ability.

**FIGURE 10:**
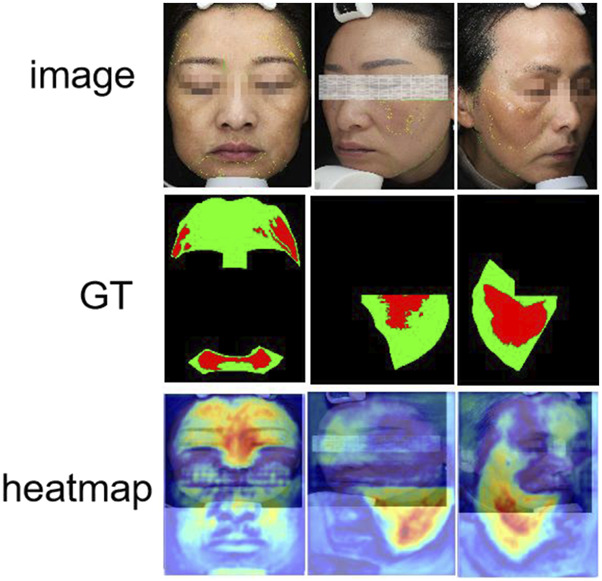
Results of the model’s thermogram visualization of the melasma region.

Moreover, to visualize the feature areas that the model may focus on, we visualized the output features of the model in the final stage. Figure 10 visualizes the heatmaps generated from the final output features, highlighting the regions that the model focuses on during prediction. Most high-response regions (in red) are concentrated in clinically relevant areas, such as the cheeks and zygomatic bones, which are common sites of melasma occurrence. This demonstrates that HHBSNet not only achieves accurate segmentation but also aligns with dermatological knowledge, thereby enhancing interpretability. The heatmaps reveal that the model effectively captures both localized lesions and diffuse patterns, maintaining robustness against variations in skin tone and illumination.

**FIGURE 11:**
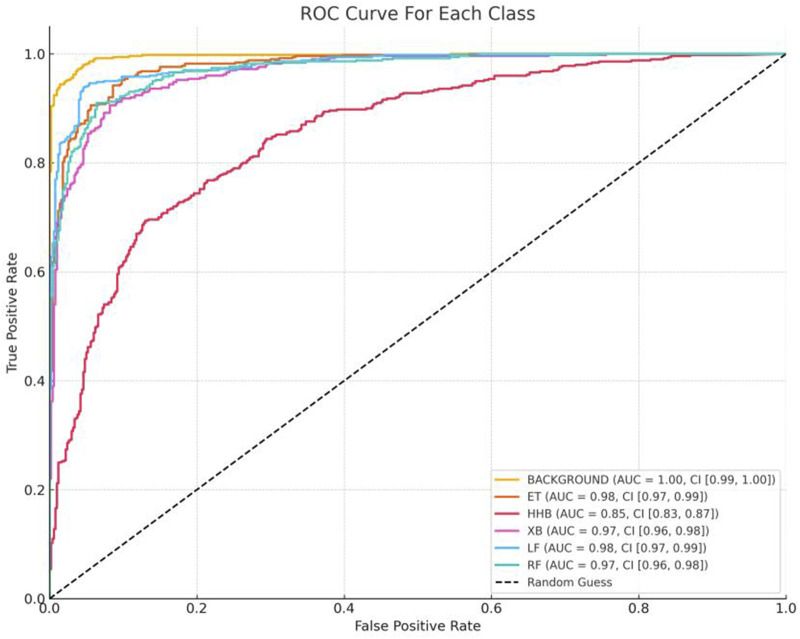
ROC curves for each category. For the six categories in the test set (BACKGROUND, ET, HHB, XB, LF, RF), the One-vs-Rest strategy was used to draw ROC curves, with the horizontal axis being the False Positive Rate (FPR) and the vertical axis being the True Positive Rate (TPR). The dotted line below the curve indicated the random classification baseline (AUC = 0.5), and the degree of deviation of the curves of each category from the baseline intuitively reflected the model differentiation ability.

Together, Figures 9, 10 complement the segmentation comparisons in Figure 8 by confirming that HHBSNet performs well across lesion categories, maintains consistent recognition under complex conditions, and provides clinically meaningful visual explanations of its predictions. Meanwhile, as can be seen in Figure 11, except for the category of HHB (melasma), the AUC (Han et al., 2024) values of the other categories are all above 0.90, which shows that the model has a high recognition accuracy in the categories of BACKGROUND, ET, XB, LF, RF, etc. The AUC of HHB is 0.85, which suggests that the model has a certain degree of error in segmenting the area of melasma, which is probably related to the fact that melasma is characterized by a large number of color distributions, blurred boundaries, and individual differences.

### 4.7 Ablation experiment

**FIGURE 12:**
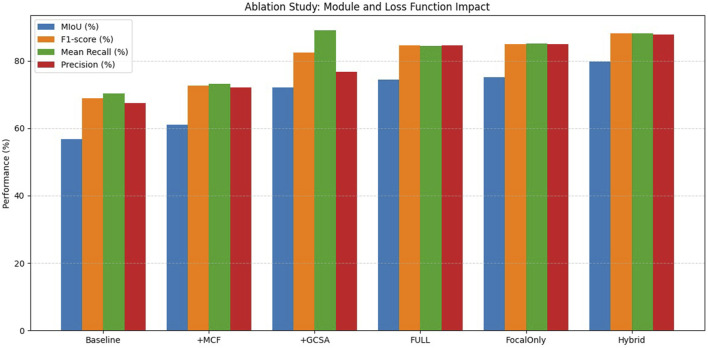
Comparative visualization of ablation experiment results.

For the purpose of evaluating the respective contributions of the proposed modules and loss functions for melasma segmentation in our HHBSNet framework, we performed a series of ablation experiments. The results of the experiments are shown in Table 4 and Figure 12 below. The baseline model, which excludes any enhancement modules and uses the basic loss function, has an average intersection over union (MIoU) rate of 56.79%, an F1 score of 68.90%, and an overall accuracy of 91.04%. After integrating the MFA module, the MIoU increases to 61.05%, indicating that MFA helps to enhance spatial features and improve segmentation quality. The GCSA module itself shows significant performance improvement, with MIoU increasing to 72.05% and F1 score increasing to 82.37%. This demonstrates the effectiveness of the GCSA module in capturing global contextual relationships, which is essential for dealing with the blurred boundaries often seen in melasma lesions.

**TABLE 4:** Ablation study results on melasma segmentation.

Model	MIoU (%)	ACC (%)	F1 (%)	Mean recall (%)	Precision (%)	Description
Baseline	56.79	91.04	68.90	70.36	67.51	Baseline without modules, basic loss
+MCF	61.05	91.25	72.66	73.14	72.11	Introducing MFA module
+GCSA	72.05	94.70	82.37	88.98	76.68	Introducing GCSA module
FULL	74.38	95.46	84.56	84.43	84.61	Full model with all modules
FocalOnly	75.09	95.58	85.00	85.11	84.89	Only using Focal Loss
Hybrid	79.69	96.68	88.10	88.18	87.80	Hybrid loss, best performance

After combining the MFA and GCSA modules (FULL model), the performance continued to improve, reaching 74.38% for MIoU and 84.56% for F1 score. Furthermore, to investigate the impact of the loss function, we replaced the standard loss with the Focal Loss alone, resulting in an MIoU of 75.09% and an F1-score of 85.00%, slightly better than the standard loss, which highlights the importance of addressing category imbalance in melasma segmentation. The best performance was obtained using a hybrid loss combining cross-entropy and focal loss (hybrid model) with an MIoU of 79.69%, an F1-score of 88.10%, and an accuracy of 96.68%. This confirms that module design and loss function selection are crucial for improving segmentation results. The experimental results clearly demonstrate the complementary advantages of the proposed module and loss designs, validating the robustness and effectiveness of our HHBSNet framework.

## 5 Conclusion

In this paper, we propose a lightweight deep neural network, HHBSNet, specifically designed for melasma segmentation. The model enhances feature extraction and lesion delineation through innovative modules. In particular, the GCSA module integrates channel attention, channel shuffling, and spatial attention to strengthen global dependency modeling of lesion areas, while the MCF module expands the receptive field without resolution loss via multi-rate dilated convolutions, thereby improving contextual feature capture. The integration of global average pooling further allows the network to fuse global semantics with local structural details, leading to more accurate recognition of blurred boundaries and irregular lesion regions. To alleviate class imbalance, we jointly adopt Cross-Entropy Loss and Focal Loss, which stabilizes training and improves recognition of minority classes.

Extensive experiments conducted on a clinical melasma dataset demonstrate that HHBSNet achieves state-of-the-art performance. Compared with competitive baselines, HHBSNet consistently attains the best scores across key metrics, with MIoU of 78.89% ± 0.16, ACC of 96.59% ± 0.03, Precision of 87.77% ± 0.13, Recall of 87.81% ± 0.08, and F1-score of 87.78% ± 0.09. These improvements are not only significant in magnitude but also stable across five independent runs, as confirmed by the small standard deviations. Notably, HHBSNet shows balanced precision and recall, ensuring accurate lesion boundary detection without compromising sensitivity.

Visualization results further confirm that HHBSNet is effective in capturing complex lesion patterns, including diffuse pigmentation and irregular boundaries, under varying illumination and skin tones. The combination of superior accuracy, robustness, and stability underscores the model’s clinical adaptability and real-world deployment potential. Future work will explore further refinements of HHBSNet’s architecture to extend its applicability to other dermatological segmentation tasks and support computer-aided diagnosis. In addition, we plan to validate the model on external public datasets (e.g., ISIC, Derm7pt) and newly collected multi-center clinical datasets, which will further assess its generalizability across diverse populations and imaging conditions.

## Data Availability

The raw data supporting the conclusions of this article will be made available by the authors upon reasonable request.
